# *Sxl*-Dependent, *tra/tra2*-Independent Alternative Splicing of the *Drosophila melanogaster* X-Linked Gene *found in neurons*

**DOI:** 10.1534/g3.115.023721

**Published:** 2015-10-26

**Authors:** Xia Sun, Haiwang Yang, David Sturgill, Brian Oliver, Leonard Rabinow, Marie-Laure Samson

**Affiliations:** * Univ Paris-Sud, Centre de Neurosciences Paris-Sud, UMR 8195, Orsay, 91405 France; † CNRS UMR 8195, Univ Paris-Sud, Orsay, 91405 Cedex, France; ‡ National Institute of Diabetes and Digestive and Kidney Diseases, National Institutes of Health, 50 South Drive, Bethesda, Maryland 20892; § Program in Computational Biology, Bioinformatics, and Genomics, University of Maryland, College Park, Maryland 20742

**Keywords:** *Sex lethal*, *found in neurons*, alternative splicing, sex-specific regulation

## Abstract

Somatic sexual determination and behavior in *Drosophila melanogaster* are under the control of a genetic cascade initiated by *Sex lethal* (*Sxl*). In the female soma, SXL RNA-binding protein regulates the splicing of *transformer* (*tra*) transcripts into a female-specific form. The RNA-binding protein TRA and its cofactor TRA2 function in concert in females, whereas SXL, TRA, and TRA2 are thought to not function in males. To better understand sex-specific regulation of gene expression, we analyzed male and female head transcriptome datasets for expression levels and splicing, quantifying sex-biased gene expression via RNA-Seq and qPCR. Our data uncouple the effects of *Sxl* and *tra*/*tra2* in females in the-sex-biased alternative splicing of head transcripts from the X-linked locus *found in neurons* (*fne*), encoding a pan-neuronal RNA-binding protein of the ELAV family. We show that FNE protein levels are downregulated by *Sxl* in female heads, also independently of *tra/tra2*. We argue that this regulation may have important sexually dimorphic consequences for the regulation of nervous system development or function.

The *Sxl* gene of *Drosophila* encodes an RNA-binding protein controlling sex determination in the soma. Initiation of *Sxl* expression in females is dependent on the number of X chromosomes in each cell (Erickson and Quintero 2007), whereas maintenance is by autoregulatory splicing of *Sxl* pre-mRNA by SXL protein (González *et al.* 2008). Female-specific *Sxl* transcripts encode a 354 AA functional protein. Male-specific *Sxl* transcripts include an additional exon introducing a stop codon in the open reading frame. The male-specific transcript has no known function, but in theory could encode a truncated SXL isoform.

The splicing regulatory cascade activated in XX females by female-specific SXL controls the splicing of *Sxl*’s own transcripts as well as those of *transformer* (*tra*). This latter gene is predicted to produce a protein with 197 AA in females and 36 residues in males, with only 13 AA in common at the N-terminus. *tra* is crucial for normal female somatic sex determination and behavior, again with no documented functions in males. TRA functions in partnership with TRA2, which also encodes an RNA-binding protein. Alternative TRA2 isoforms of 226 and 264 AA are present in the soma of both females and males (Mattox *et al.* 1990). As for TRA, no known function in male somatic tissues has been reported for TRA2, although it does function in the male germline (Unni *et al.* 2003). The absence of either TRA or TRA2 protein eliminates female-specific alternative splicing of *doublesex* (*dsx*) and *fruitless* (*fru*) transcripts, which encode transcription factors essential for sex-specific development and behavior. SXL also downregulates the expression of *male-specific-lethal-2* (*msl2*) through alternative splicing and translation (Valcárcel *et al.* 1993; Bashaw and Baker 1995; Kelley *et al.* 1997; Gebauer *et al.* 1998; Graindorge *et al.* 2013). This gene encodes a regulator of chromatin binding factors thought to be functional only in males. MSL2 is key to the normalization of gene expression between the single X chromosome of the males and the two copies of females, a phenomenon referred to as dosage compensation. The prevalent view is that dosage compensation in *Drosophila* relies on increased X-linked transcription in XY males by protein factors absent in XX females, a key player being *msl-2* (Conrad and Akhtar 2012), although alternative mechanisms, including the repression of X-linked genes in females, have been proposed (Kelley *et al.* 1995; Birchler *et al.* 2011).

Analyses via microarrays, SAGE (Serial Analysis of Gene Expression), or RNA-Seq suggest that hundreds of genes are differentially expressed in male and female heads and/or are regulated by *tra* or *dsx* (Arbeitman *et al.* 2004; Goldman and Arbeitman 2007; Chang *et al.* 2011), see Samson and Rabinow 2014 for review, but only a small number of loci have been validated by further experiments.

Sex-specifically expressed genes directly regulated by the somatic sex determination pathway include the yolk protein genes *Yp1* and *Yp2*, as shown via transgenic constructs and DNA-binding experiments with DSX protein (Coschigano and Wensink 1993). Another gene activated by direct DSX-F binding to its promoter region is *Fad2/desatF*, encoding a fatty acid desaturase essential for pheromone biosynthesis in female oenocytes (Chertemps *et al.* 2007; Shirangi *et al.* 2009). *Bab1* expression, which regulates abdominal pigmentation, is activated in females by direct binding of DSX-F and the transcription factor ABD-B, whereas it is repressed in males by binding of DSX-M (Williams *et al.* 2008). Few other targets have been validated for regulation by DSX, TRA, or TRA2. Among those loci is *eloF*, a gene involved in long-chain hydrocarbon biosynthesis, specifically expressed in female carcasses under *tra* control (Chertemps *et al.* 2007) .

Sex-biased expression of the genes *CG11458*, *yellow-c*, *CG7433*, and *Sodh-1* was reported in heads and validated (Fujii and Amrein 2002). In adult neurons, transcripts from the noncoding genes *roX-1* and *roX-2* (*RNA on the X*) were also found to be specifically expressed in males (Amrein and Axel 1997). Other examples of sex-specifically expressed or biased transcripts include *turn on sex-specificity* (*tsx*) and *sex-specific enzyme 1* and *2 (sxe1*, *sxe2*), which are under *tra/tra2* control in heads (Fujii and Amrein 2002; Fujii *et al.* 2008) as well as *cpn (calphotin)*, expressed at higher levels in males and regulated by *dsx* (Goldman and Arbeitman 2007). The *neuropeptide F* (*npf*) gene is expressed at higher levels in male relative to female heads and is negatively regulated by *tra* (Lee *et al.* 2006). Finally, sex-specific expression of *fit* (*female-specific independent of transformer*) in heads has been reported as independent of *tra* and *tra2* (Fujii and Amrein 2002), but others reported it to be *tra*-dependent (Evans and Cline 2013).

In addition to sex-specific differences in expression levels, sex-biased splicing has also been reported in fly heads and validated by qPCR (Telonis-Scott *et al.* 2009; Hartmann *et al.* 2011; Sturgill *et al.* 2013). Given that SXL, TRA, and TRA2 encode splicing regulators, some of this sex-specific/biased alternative splicing may be direct, unless it occurs via other RNA-binding proteins regulated by the transcription factors FRU and DSX. Sex-biased/sex-specific expression of alternative transcripts was reported for *bcd* (*bicoid*), *squid/hrp40*, *P-element somatic inhibitor* (*Psi*), *Hrb27c* (*Heterogeneous nuclear ribonucleoprotein at 27C*), *Rbp2* (*RNA binding protein 2*), and *BicC (Bicaudal C*), all encoding proteins that bind RNA with potential roles in posttranscriptional regulation (Telonis-Scott *et al.* 2009; Hartmann *et al.* 2011). Alternative RNA-binding protein isoforms encoded by these transcripts thus might alter sex-biased splicing of target genes, although whether these differences are under control of the canonical sex determination hierarchy remains unknown.

Additional transcripts showing evidence of sex-biased splicing in heads include those from the *J domain containing protein* (*jdp*) gene (Hartmann *et al.* 2011). Sex-biased/sex-specific alternative splicing for transcripts of *jigr1* (*jing interacting gene regulatory 1*: transcription factor), *exba*/*krasavietz* (ribosome binding), *CCR4/twin* (protein binding/negative regulation of translation), *LIMK1* (*LIM-protein kinase1*), *Ubi-p63E* (ubiquitin homeostasis), and *unc-115* (Zinc finger, actin binding) was also reported.

To gain further insight into regulated sexual dimorphism of gene expression, we used a head RNA-Seq dataset as a starting point (Sturgill *et al.* 2013). We identified sex-biased events and performed qPCR to determine if sex-biased/sex-specific gene expression was controlled by the regulators of the canonical somatic sex determination pathway *tra*, *tra2*, or *Sxl*. Our results identify the X-linked gene *found in neurons* (*fne*) as a target of *tra*-independent, *Sxl*-dependent regulation in heads. Direct analysis of FNE protein levels reveals that SXL, but not TRA or TRA2, not only regulates the splicing of *fne* transcripts but also the level of FNE protein in female heads.

## Materials and Methods

### *Drosophila* methods

Flies were maintained on standard yeast/corn meal media at 25°. The isogenic *w^1118^* Canton-S (B) stock was used to generate wild-type RNA (Edwards *et al.* 2006; Yamamoto *et al.* 2008). Details on fly husbandry used for the RNA preparations were previously described (Sturgill *et al.* 2013). Stocks were obtained from the Kyoto *Drosophila* Genetic Resource Center (KY), Bloomington *Drosophila* Stock Center (BL), B. Baker (BB), T. Cline (TC), and W. Mattox (WM): *Dp(1;Y)B^S^/w^a^*; *st^1^ tra^1^/TM2*, *Ubx^130^ e^s^* (KY), *w^1118^*; *tra^1^/TM3*, *Sb* (derived in our lab from *w^a^*; *tra^1^*/*TM2*, BL), *Dp*(*1;Ybb^−^*)*B^S^*; *cn^1^ tra2^B^ bw/CyO* (KY), *y w*/*Dp*(*1;Y*)*B^S^*; *tra2^B^*/*CyO* (WM), *Df(2R)trix*/*CyO* (BL), *y*/*Yy^+^*; *tra2^1^/SM1* (BB), *y w Sxl^M1,fΔ33^ ct^6^ sn^3^/ Binscy* (tc), *y cm Sxl^f7M1^ ct v* /Y; *P(Sxl^+^w^-^)9A/+* (TC). We generated *w^+^*; *st^1^ tra^1^/T(2;3) CyO TM1* and *w^+^*; *cn^1^ tra2^B^ bw^1^/CyO* males and crossed them to females from the appropriate stocks to generate sibling XX and XY flies, homozygous and heterozygous mutants for *tra* and *tra2*, respectively. More detailed information and the *Sxl* genotypes are found in the relevant figure legends.

### Head RNA preparation, RNA-Seq, and quantitative RT-PCR

Methods were previously described (Sturgill *et al.* 2013). RNAseq data were downloaded from NCBI Sequence Read Archive (GSM928376, GSM928377, GSM928383, GSM928384, GSM928392, and GSM928393). These include data from two biological replicates yielding ≥200 million mapped reads for male and for female head samples, documenting the expression of 17,142 loci in FlyBase release 5.57 (St Pierre *et al.* 2014). The downloaded sra files were converted to fastq by sratoolkit (2.4.2-1) with the command line: fastq-dump–split-3. Reads that belong to the same biological replicate but different technical replicates were merged first, and then were uniquely mapped back to the genome using TopHat (2.0.10) (Trapnell *et al.* 2009) and Bowtie2 (2.1.0) (Langmead and Salzberg 2012) with the following settings: -g 1–library-type fr-firststrand –G. The output bam files were then indexed and sorted by samtools (0.1.19) (Li *et al.* 2009) and used in Spanki (0.4.3) (Sturgill *et al.* 2013) and MISO (0.5.2) (Katz *et al.* 2015) for alternative splicing analysis. For Spanki analysis: (1) we ran with the built-in command spankijunc from Spanki with “-m all” option; (2) curated junctions were built from all the junction files made by the first step; (3) the command merge_jtabs was run from Spanki to pools all biological replicates together; (4) splicing events were generated from annotations by Astalavista (Foissac and Sammeth 2007) with the following options: -c asta +ext; (5) the built-in command spankisplice from Spanki was run to make splicing events from junctions with support of the Astalavista output; and (6) the built-in command splicecomp from Spanki was run to compare the alternative splicing between female and male heads. We calculated *P* values from Fisher’s exact test and used FDR (corrected by Benjamini-Hochberg) <0.05 as the cutoff. More details can be found in the Supporting Information of Sturgill *et al.* (2013). MISO was used to visualize the alternative splicing pattern for the raw bam files as follows: (1) we merged the bam files with different biological replicates together to make two large bam files for female and male, respectively; (2) we used gtf2gff3.pl (http://genes.mit.edu/burgelab/miso/scripts/gtf2gff3.pl) to convert the gtf annotation to gff3 and then used the built-in command index_gff from miso to index the gff3 annotation; (3) we ran MISO with the merged bam files with the indexed annotation from the previous step and the option:–read-len 76; (4) we obtained the mapped read number from bam files by samtools view –c; (5) we ran the command sashimi_plot from MISO with the following settings: scale of intron and exon = 1:1, ymax = 56, and the total mapped read information from the previous step; and (6) colors and fonts of the sashimi_plot were further modified by Adobe Illustrator (CC 2014) and coding position was guided by Integrative Genomics Viewer (IGV 2.3.46).

### Sequencing of *fne* cDNA

To verify the Flybase model that associates the 5′ alternatively regulated exons in event ASTA0020150 (Supporting Information, File S1) to the *fne* ORF, we sequenced *fne* cDNAs from male and female heads. Our data (Genbank accession numbers: KJ815141, KJ815142) support the Flybase *fne* gene model, both in terms of exon structure, with individual cDNAs including both 5′ exons and the *fne* ORF, and for the identity of the coding strand (GT… AG intronic splice sites).

### qPCR Statistics

Comparisons of stable transcript levels were performed with STATISTICA software (StatSoft, Inc.). Male/female comparisons were performed by *t*-test, and *P* values were corrected for multiple testing using the method of Benjamini-Hochberg with InVivoStat software (http://www.bap.org.uk/invivostat.php), a gift from S. Bate (Clark *et al.* 2012). Transcript levels in multiple genotypes were analyzed by two-way ANOVA, followed by a post-hoc test with the Scheffé method.

### Statistics for the enrichments

Enrichments are defined as [abundance of transcript form 1 / transcript form 2] in one genotype relative to [abundance of transcript form 1 / transcript form 2] in CS females. We compared [abundance of transcript form 1 / transcript form 2] in one genotype relative to either CS females [abundance of transcript form 1 / transcript form 2] or CS males [abundance of transcript form 1 / transcript form 2]. Raw transcript abundance values were used to calculate C.I.s and *P* values for those ratios of transcript levels, as in Fay and Gerow (2013). We used their spread sheet and adapted it to our data set. Bonferroni corrections of the *P* values were implemented when more than one comparison was performed using the data set (Figure 3, Figure 4)

### Head protein preparations and FNE immunodetection

Frozen fly heads (30–100) were homogenized on ice in 40 μl of freshly prepared 1X PBS, 0.5% IGEPAL CA-630, 1 mM EGTA pH 8, 0.5 μg/μl leupeptin, 1 μg/μl peptastin, and 0.2 mM PMSF. Samples were spun for 10 min, 10,000 rpm, at RT and the soluble proteins were recovered in a fresh tube. Protein extracts (four head-equivalents per lane) were resolved on 10% SDS-polvacrylamide and transferred to a PVDF membrane (0.45 μm; Millipore Corporation). Immunodetection was performed at room temperature as follows: (1) blocking in 5% skim milk, TBST (50 mM Tris; 0.15 M NaCl; 0.05% Tween-20; pH 7.6) 30 min, 37°; (2) incubation with 1:1000 rat polyclonal anti-FNE (Zanini *et al.* 2012) in TBST for 30 min; (3) three washes in TBST, 10 min each; (4) incubation with 1:5000 anti-rat IgG (Invitrogen) in BST for 30 min; and (5) three washes in TBST, 10 min each. Detection was performed with chemiluminescent reagents (Immobilon Western, Millipore) and quantification was on a Carestream (In-Vivo FPRO) using the Carestream MI software.

### Data availability

Figure S1 is a graphic representation of sex-biased alternative splicing for key splice events. File S1 is the complete Spanki analysis of the RNA-Seq data. File S2 and File S3 are detailed statistical analyses, respectively for qPCR results and for enrichments. File S4 is a supplemental discussion. Table S1 contains the Spanki analysis of the RNA-Seq data and qPCR validation for key splice events. Table S2 provides details on the primers. Accession numbers for sequence and gene expression data are specified in the *Materials and Methods*.

## Results

### Identification of genes whose alternative splicing patterns differ between female and male heads

Analysis of RNA-Seq replicates from female and male heads prepared from an isogenic *w^1118^* Canton-S (B) stock (Edwards *et al.* 2006; Yamamoto *et al.* 2008), referred to as CS throughout this article, was previously reported (Sturgill *et al.* 2013). We remapped the same reads to the current genome annotation (FlyBase 5.57) (St Pierre *et al.* 2014) with updated versions of RNAseq analysis software (see *Materials and Methods*) that provide candidate gene lists for further study (File S1).

**Figure 1:**
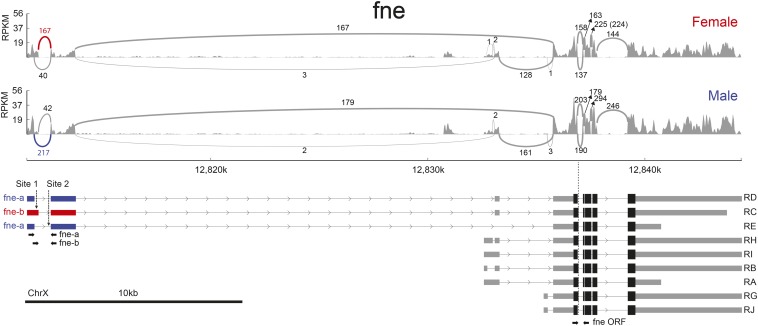
Graphic representation of sex-biased alternative splicing for *fne* in the RNA-Seq analysis. RPKM for each alignment track are shown as sashimi plots (Katz *et al.* 2015). Arcs denote splice junctions, quantified in spanning reads, as specified near each exon junction. Exons and splice junctions relevant to the sex-biased splice event are in red (female-enriched junction) or blue (male-enriched junction). The nine transcripts predicted by the FlyBase (5.57) gene model are shown at the bottom. We have sequenced cDNAs that link the 5′ alternatively regulated exons to the *fne* ORF (see *Materials and Methods*). X chromosome coordinates are indicated and arrows in introns represent the direction of transcription. The structure of the RT-PCR amplicons specific for *fne-a*, *fne-b*, and *fne* ORF, respectively, are shown with converging arrows below the transcript diagrams. The position of the stretches UUUUUUAUCUCUUUUU (site 1) and UUUUUUUU (site 2) mentioned in File S4 are shown with vertical arrows.

Among the genes identified were *Sxl*, *dsx*, and *fru*, which produce alternatively spliced components of the sex determination pathway (Figure S1). A new sex-biased splicing event was identified in *fne* (Figure 1). The analysis of splicing by RNA-Seq has many caveats of statistical power, and there are still serious alignment errors that make accurate assessment of splice junctions problematic (Sturgill *et al.* 2013). For instance, in our analyses, the transcripts of *tango13* apparently display sex-specific splicing but their computed sex-biased expression is not deemed statistically significant (Figure S1). However, theses transcripts were independently identified as sex-specifically spliced in a microarray analysis (McIntyre *et al.* 2006). Therefore, we also decided to further examine the splicing of *tango13* transcripts.

**Figure 2:**
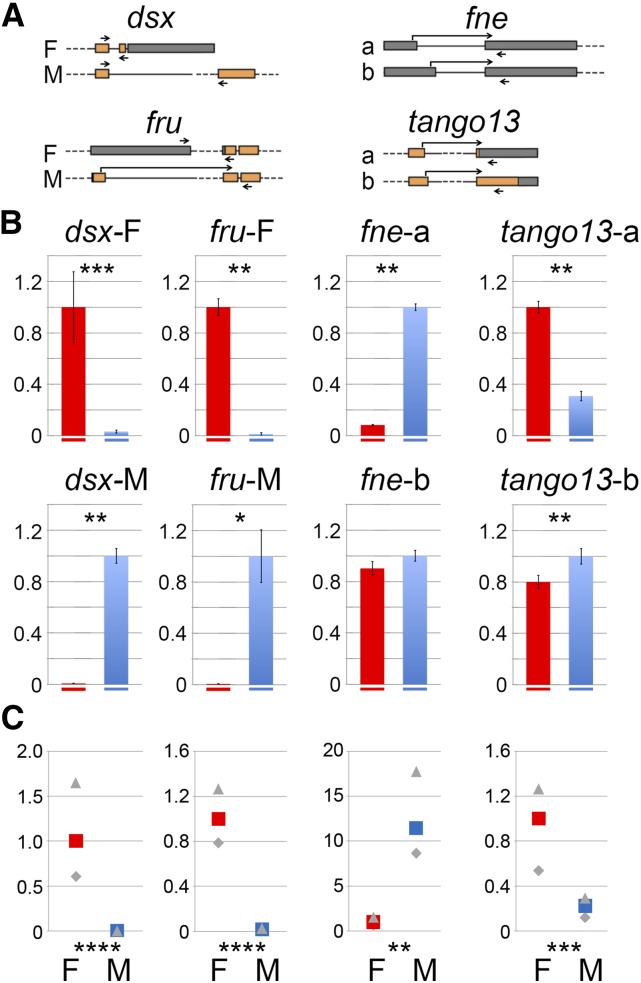
Sex-dependent alternative splicing of *dsx*, *fru*, *fne*, and *tango13*. (A) Diagrams of the qPCR tested sex-biased alternative splicing events. Exons are represented by boxes; orange indicates coding regions and gray indicates untranslated regions (UTRs). Introns are represented by lines (in scale) and dashes (out of scale). The positions of the primers used for qPCR analysis are indicated by arrows joining two exons when a primer overlaps an exon junction. (B) qPCR analysis of four alternative splicing events. The bar plots show the female (red) *vs.* male (blue) relative transcript levels as determined by qPCR. *P* values (*t*-test) for the comparison of transcript abundance in female *vs.* male are indicated on each panel. *P ≤ 0.05, **P ≤ 0.01, ***P ≤ 0.001, ****P ≤ 0.0001 (see File S2). (C) As a convenient way to compare splicing between two genotypes, we use enrichment values. The average enrichments are computed from our qPCR quantifications as [abundance of transcript form a / transcript form b] in one genotype relative to [abundance of transcript form a / transcript form b] in CS females. By definition, the enrichment is 1 in CS female. Maximum and minimum enrichments calculated from our qPCR quantifications are indicated in gray. The complete statistical analysis of enrichments is in File S3 (Fay and Gerow 2013). *P* values for the comparisons of enrichments are indicated below the bar plots (*, **, ***, and **** as above).

We used qPCR to explore these alternative events and confirmed significant sex differential splicing for all of them (Figure 2, Table S1). Although we measured individual transcript levels, we focused on the changes in the relative enrichment of one alternative splice form *vs.* the other form when comparing genotypes, as detailed in the legend of Figure 2. The major advantage of using enrichment in the analysis of splicing is that it frees the data from the potential impact of different backgrounds on transcript expression levels, facilitating comparisons between genotypes.

Importantly, both RNA-Seq and qPCR identified and quantified the sex-specific alternative *dsx* and *fru* transcript isoforms (Figure 2 and Figure S1), demonstrating that known alternative splicing events were captured in the datasets. We also quantified with qPCR the sex-biased alternative splicing events in *fne*, obtaining congruence between RNA-Seq and qPCR results where two different 5′ donor sites and a fixed 3′ acceptor site in the 5′ UTR are used differentially in males and females (Figure 2, Table S1): RNA-Seq computes a 21-fold enrichment in the relative amounts of *fne-a vs. fne-b* in males compared to females, whereas qPCR measures a 9- to 18-fold increase (*P* ≤ 0.01) (Figure 2). In the case of *tango13*, qPCR, but not RNA-Seq (Spanki analysis), measured significant gene-level sex-biased expression (Table S1). Analysis of the *tango13* transcripts reveals the use of alternative 5′ splicing donor sites predicted to shift the reading frame. As a result, two forms of TANGO13 with alternative carboxy termini and different sizes would be produced. Transcripts encoding the predicted 346 AA isoform were predominant in females, whereas those encoding the 499 AA isoform were predominant in males (Figure 2). qPCR measures up to a five-fold increase (*P* ≤ 0.001) (Figure 2) in the enrichment of *tango13-b vs. tango13-a* in males compared to levels observed females.

### Differential impact of *tra2^1^* and *tra2^B^* alleles on the splicing of *fru* but not of *dsx*

To better understand the modalities that govern sex-specific splicing, we quantified the potential dependence of the sex determination pathway on the sex-biased splicing events. We used the following alleles: *tra^1^*, which deletes approximately 1 kb of the gene, including the entire ORF (Yuan and Belote 1995); *tra2^1^*, which is a spontaneous allele with an unknown lesion; and *tra2^B^*, which introduces a premature stop codon and is predicted to produce a truncated polypeptide missing a portion of the RNA recognition motif (RRM) and the RS2 domain essential for TRA2 function (Mattox and Baker 1991). However, suppression of the stop codon cannot be excluded because it is a common occurrence in *Drosophila* (Jungreis *et al.* 2011). XX females homozygous for *tra^1^* or *tra2^B^* or that are *tra2^1^*/*Df(2R)trix* exhibit a full cuticular transformation of female to male-like (pseudomales).

**Figure 3:**
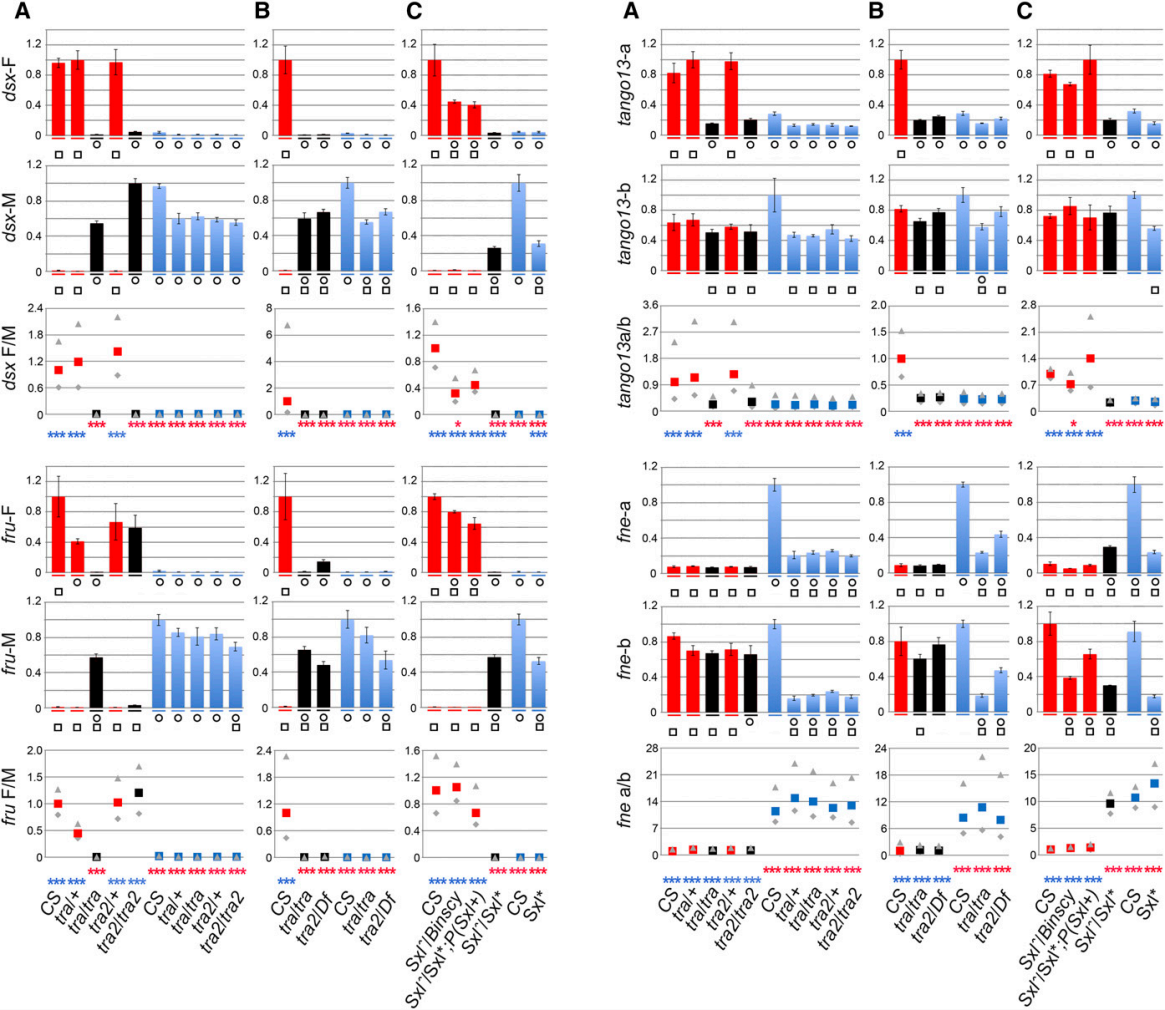
Impact of *tra*, *tra2*, and *Sxl* mutations on alternative splicing. The bar plots show relative transcript levels as determined by qPCR in females (red), males (blue), and pseudomales represented in black. The primers used for qPCR are specified in Figure 2A and Table S2, and genotypes are indicated below the bar plots. Heterozygous and homozygous flies mutant for *tra*, *tra2*, and *Sxl*, respectively, are siblings. They were respectively obtained from the cross between female *w^1118^*; *tra^1^/TM3 Sb* and male *w^+^*; *st tra^1^/T(2;3) CyO TM1*, from the cross between female *y w*/*y w*; *tra2^B^*/*CyO* and male *w^+^*; *cn tra2^B^ bw/CyO*. *tra2^1^/Df(2R)trix* and from the cross between female *y*/*Yy^+^*; *tra2^1^/SM1* and male *Df(2R)trix*/*CyO*. Siblings of heterozygous and homozygous *Sxl* mutants are generated from crosses between female *y w Sxl^M1,fΔ33^ ct^6^ sn^3^/Binscy* and male *y cm Sxl^f7M1^ ct v*/Y; *P(Sxl^+^w^-^)9A/+* or *+*/*+*. Conclusions of the ANOVA (two-way, followed by post hoc test): [ANOVA: F(32, 86.415) = 33.076, *P* ≤ 1E-5] for the experiment in (A); [F(16, 22) = 26.695, *P* ≤ 1E-5] for the experiment in (B); and [F(40, 42.025) = 56.186, *P* ≤ 1E-5] for the experiment in (C) are provided under each set of bar plots. Circles (squares) denote statistically significant departure from CS female (male) expression (as detailed in File S2). Enrichments are defined and analyzed as specified in the legend of Figure 2. The conclusions of the statistical analysis (after Bonferroni correction, see File S3) are shown in red for comparisons with CS females and in blue for comparisons with CS males. *P ≤ 0.05, **P ≤ 0.01, ***P ≤ 0.001.

Consistent with the current sex determination model, we found that in these three types of XX pseudomales the major *dsx* transcript form is male-specific *dsx*-M and not female-specific *dsx-F* (*P* ≤ 1E-6) (Figure 3). Based on the qPCR, *dsx-F* enrichment relative to *dsx-M* in these XX pseudomales is reduced at least 600 compared to CS females (*P* ≤ 1E-3) (Figure 3), not significantly different from the level observed in CS males (*P* ≥ 0.05) (Figure 3). Interestingly, although in *tra^1^*/*tra^1^* and *tra2^1^*/*Df(2R)trix* XX pseudomales *fru* is alternatively spliced in a male mode (*P* ≤ 1E-3), *tra2^B^*/*tra2^B^* pseudomales produce a major *fru-F* isoform similar to CS females (*P* > 0.05) (Figure 3): *fru-M* enrichment relative to *fru-F* in *tra2^B^*/*tra2^B^* pseudomales is 0.6- to 1.2-fold the levels observed in CS females, but it is at least 40-fold higher in the other pseudomales. We conclude that in the context of limited *tra2* function (in *tra2^B^* mutant XX flies transformed from females to pseudomales) *dsx* transcripts are spliced in a male mode but *fru* transcripts are spliced in female mode. This suggests that the effect of the homozygous *tra2^B^* mutations on *fru* splicing is weaker than the effect of homozygous *tra^1^*/*tra^1^* and *tra2^1^*/*Df(2R)trix*. Although we cannot exclude that the *tra2B* background is responsible for unexpected splicing of the *fru* alternative transcripts, we suggest that these observations may reveal distinct dosage requirements for TRA2 in the regulation of *dsx* and *fru* splicing, respectively.

### Regulation of *tango13* alternative splicing events in the heads of *tra*, *tra2*, and *Sxl* mutant females

Because sex-biased expression, and perhaps alternative splicing, is expected to be regulated by the canonical sex determination pathway, we addressed the level of control of *tango13* within the sex determination network by examining its splicing via qPCR in XX females, XX pseudomales, and XY males (Figure 3). Specifically, we used *tra^1^*, *tra2^B^*, *tra2^1^*/*Df(2R)trix* and also a heteroallelic combination of the hypomorphic alleles *Sxl^M1,fΔ33^* and *Sxl^f7M1^* (Evans and Cline 2013). *Sxl^M1,fΔ33^* / *Sxl^f7M1^* females are phenotypically masculinized to the same degree as other reported *Sxl* mutant combinations but are more viable and longer lived (Evans and Cline 2013). To ascertain that splicing changes were specifically due to *Sxl*, we compared these pseudomales to sibling female *Sxl^M1,fΔ33^* / *Sxl^f7M1^*, *P(Sxl^+^w)9A*, where the *Sxl* mutation is rescued with one copy of an autosomal *Sxl^+^* minigene. As expected, we found that *dsx-M* and *fru-M* become the major transcript forms in the *Sxl*^-^ XX pseudomales. Based on qPCR, *dsx-F* enrichment relative to *dsx-M* in *Sxl* XX pseudomales is decreased at least 300-fold compared to CS or *Sxl^M1,fΔ33^* / *Sxl^f7M1^*, *P(Sxl^+^w)9A* females (*P* ≤ 0.0006) (Figure 3C). Similarly, *fru-F* enrichment relative to *fru-M* in *Sxl*^-^ XX pseudomales is lowered at least 1800-fold compared to levels observed in CS females or in *Sxl^M1,fΔ33^* / *Sxl^f7M1^*, *P(Sxl^+^w)9A* females (*P* ≤ 0.0006) (Figure 3C), and is close to the levels observed in CS males (*P* ≥ 0.05) (Figure 3C). This is consistent with SXL regulating these two genes in female heads.

Using this approach, we found evidence for the misregulation of *tango13* in *tra*, in *tra2*, and in *Sxl*^-^ XX pseudomales, where *tango13* is spliced in a male mode (Figure 3). Compared to CS females, the enrichment of *tango13-b vs. tango13-a* transcript levels increased up to seven-fold in XX homozygous *tra^1^* and *tra2^B^* and in XX *tra2^1^*/*Df(2R)trix* and *Sxl^M1,fΔ33^* / *Sxl^f7M1^* pseudomales (*P* ≤ 0.0006) (Figure 3), close to male levels (*P* > 0.05). Noticeably, this happens while the level of *tango13-b* remains constant (*P* ≥ 0.18). This is an unexpected outcome for a pair of mutually exclusive transcripts whose expression was anticipated to be modified in a reciprocal manner. These data are consistent with *tango13* sex-biased regulation of gene expression by *tra*/*tra2* at a level distinct from alternative splicing, perhaps via DSX and/or FRU or further downstream in the pathway.

### Regulation of *fne* alternative splicing in *Sxl* but not in *tra* or *tra2* mutant heads

Northern blot analyses of *fne* transcription patterns resolve at least four tissue-regulated and developmentally regulated transcripts (Samson and Chalvet 2003). In heads, these analyses identified two (groups of) transcripts approximately 4.4 kb long, and two more approximately 7.5 kb. The Flybase gene model predicts nine transcripts produced from two major transcription initiation sites, compatible with the sizes determined by Northern blot analysis (Figure 1). The 199 nt size difference between the *fne-a* and *fne-b* forms generated by alternative splicing in the upstream 5′ UTR is not sufficient for discrimination by Northern blot analyses. The optional sex-neutral extension of the first coding exon (six nucleotides) (Figure 1) generates two alternative *fne* transcript forms that, in conjugation with the use of three alternative 3′ UTRs (Hilgers *et al.* 2011), have the potential to generate six alternative forms of *fne-a* and *fne-b*, respectively. In head samples, the number of splice junction reads in the 5′ UTR of *fne-a* and *fne-b* (167+40 = 207 in females and 217+42 = 257 in males) (Figure 1) is comparable to the number of constitutive splice junctions in the coding *fne* region (144, 163, or 225 in females and 179, 246, or 294 in males) (Figure 1). This observation suggests that transcription initiating at the upstream 5′ UTR significantly contributes to the total *fne* RNA pool, and that the isoforms RD, RC, RE (Figure 1) represent the bulk of *fne* RNA.

Alternative splicing of *fne* in heads follows an unconventional pattern in that its sex-specific splicing is unchanged from CS females in *tra* or *tra2* mutant XX pseudomales (Figure 3) (P ≥ 0.05) and significantly differs from splicing in CS males (Figure 3) (P ≤ 0.0006). This is unexpected and in striking contrast with changes of *dsx*, *fru*, and *tango13* alternative transcripts showing quantitative differences in *tra* and *tra2* XX pseudomales. Further, the very robust female mode splicing is unchanged not only in *tra^−^* and *tra2^−^* XX pseudomales but also in a *Doa* heteroallelic combination that causes sexual transformations (not shown) due to alteration of DOA, a kinase that phosphorylates TRA and TRA2 (Du *et al.* 1998). These data demonstrate that the sex-biased alternative splicing event in the upstream 5′ UTR of *fne* transcripts is not regulated by *tra*/*tra2* in females.

Because we found that the sex-specific regulation of *fne* splicing does not require *tra* and *tra2* function, we investigated alternative possibilities. There is some evidence for alternative sex determination/differentiation pathways dependent on *Sxl* but independent of *tra* and *tra2* (Evans and Cline 2013) or independent of *Sxl* (Hartmann *et al.* 2011). We therefore examined the impact of *Sxl* mutations on *fne* alternative splicing. We found that in XX *Sxl^M1,fΔ33^*/*Sxl^f7M1^* pseudomales, *fne* splicing switches to a male mode (Figure 3). The enrichment of the *fne-a vs. fne-b* transcript in these XX pseudomales is similar to that of CS males (0.6-fold to 1.0-fold, *P* > 0.05), whereas it increases 8-fold to 12-fold compared to that in CS females (*P* < 0.0006). In contrast to the XX *Sxl* pseudomales, in both classes of our controls (*y cm Sxl^f7M1^*/*Binscy*: XX and *Sxl^M1,fΔ33^*/*Sxl^f7M1^*, *P(Sxl^+^w)9A* females), the enrichment of *fne-a vs. fne-b* transcript is close to that in CS females (less than two-fold increase, *P* > 0.05) and is distinct from that in CS males (approximately eight-fold, *P* < 0.0006), indicating that this particular *fne* splicing regulation is intact in the controls (Figure 3). Importantly, the CS female-like splicing of *fne* in XX *Sxl^M1,fΔ33^*/*Sxl^f7M1^*, *P(Sxl^+^w)9A* females confirms the specificity of the effect of *Sxl* on *fne* splicing in XX *Sxl^+^* females.

### FNE protein levels are independent of *tra* and *tra2* in the two sexes but depend on *Sxl in female heads*

**Figure 4:**
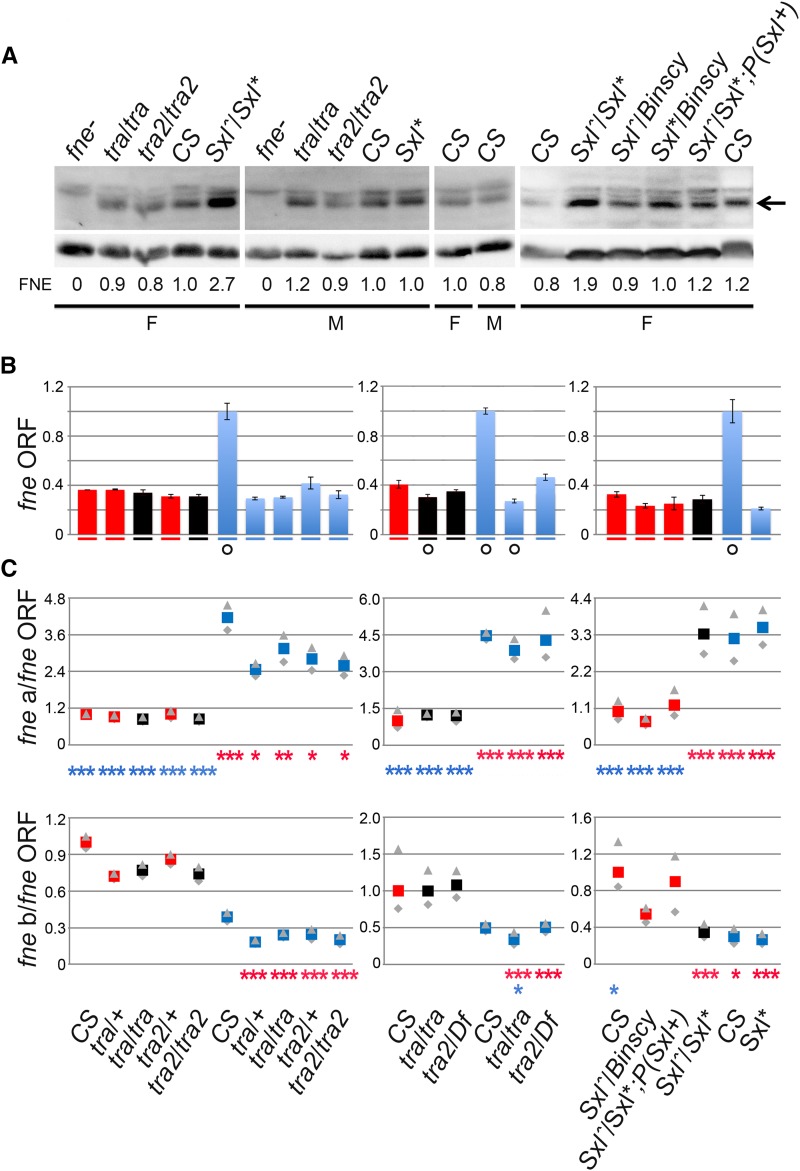
Impact of *tra*, *tra2*, and *Sxl* mutations on the expression of FNE protein and *fne* ORF-containing transcripts. (A) Immunoblot analysis of head protein extracts with anti-FNE antibodies. Tubulin is used as a loading control. Genotypes are as in Figure 2 and Figure 3. Head protein extracts were prepared from sibling flies born from the same parents and collected at the same time as the flies used for the head RNA preparations. An arrow indicates the position of FNE protein on the blots. The quantification of relative amounts of FNE is shown below the blots. (B) Quantitation of the amount of *fne* ORF. Genotypes and RNA batches used in this experiment are the same as those used for Figure 3. Each set of bar plots shows the female (red), pseudomale (black), and male (blue) relative transcript levels (determined by qPCR). Symbols summarizing the conclusions of ANOVA [F(4, 22)=107.85, *P* ≤ 1E-5 for the experiment with *tra* and *tra2* mutants, F(2, 18)=217.66, *P* ≤ 1E-5 for the experiment with *tra*, *tra2*, and *Df(2R)trix*, F(5, 18)=108.39, *P* ≤ 1E-5 for *Sxl* as detailed in File S2] followed by post hoc test are as in Figure 3. (C) Enrichments (defined in Figure 2) of *fne-a* (and, respectively, *fne-b*) relative to ORF-containing *fne* transcripts are shown below the bar plots using the same symbols as in Figure 3, and as detailed in File S3.

Due to posttranscriptional regulation, transcript levels only partially predict protein abundance, the latter being more directly relevant to function. Thus, to further evaluate the impact of *Sxl* on *fne* expression, we examined FNE protein levels in heads by immunoblot. We found that FNE levels are independent of sex in CS heads, with relative amounts in females of 0.9 *vs.* 1.0 in males (SEM = 0.119, based on four immunoblots). We found that in male and female CS, *tra^1^*/*tra^1^* and *tra2^B^*/*tra2^B^* mutants all share similar FNE protein levels (Figure 4A), confirming independence of *fne* regulation and *tra*/*tra2* function. However, FNE protein levels are increased, on average, 2.4-fold (four measures, SEM = 0.067) in XX *Sxl^M1,fΔ33^* / *Sxl^f7M1^* pseudomales compared to CS females as well as all the other genotypes. In contrast, FNE levels remain unchanged in *Sxl^M1,fΔ33^* / *Sxl^f7M1^*, *P(Sxl^+^w)9A* females (1.1-fold variation, two measures, SEM = 0.15) where the *Sxl* mutation is rescued with a minigene (Figure 4A). Our data thus reveal a perfect correlation between changes in *fne* alternative splicing in XX *Sxl*^-^ pseudomales and those in FNE protein levels beyond potential changes in levels of individual *fne-a* and *fne-b* transcripts. It is worth mentioning that the expression of ELAV, another pan neuronal protein paralogous to FNE, is unchanged in all the tested genotypes (not shown). This indicates that the increased expression of FNE in XX *Sxl^−^* pseudomales is not due to an overgrowth of neurons or the nervous system, that this effect is restricted to *fne* and is not found among all members of the family.

Because it cannot be excluded that the increased FNE protein levels in the heads of XX *Sxl^−^* pseudomales are due to an increase in the amounts of *fne* stable coding transcripts, we examined those via qPCR in all the genotypes used in this study using the primers shown in Figure 1. Aside from high levels in CS males (with no impact on FNE protein levels), no significant difference in *fne* ORF-containing RNA is detected among other males, females, and pseudomales (*P* > 0.2) (Figure 4B). Further, we found that the enrichment of *fne-a* or *fne-b* relative to the *fne* ORF RNA follows XX-specific and XY-specific patterns, except in XX *Sxl^M1,fΔ33^*/*Sxl^f7M1^* pseudomales, where it departs from the CS female pattern (*P* ≤ 0.0006) and is male-like (*P* ≥ 0.05) (Figure 4C). Thus, we obtained further evidence that *fne* is regulated in a male model in the XX *Sxl^−^* pseudomales, but we see no evidence for increased *fne* transcript levels in these animals.

In summary, our data on *Drosophila* head RNA show that: (1) *fne* is differentially spliced in the two sexes; (2) *tra* and *tra2* do not regulate *fne*; (3) *Sxl* does not impact coding transcript levels but regulates the splicing of *fne*, causing a switch from the default male splicing pattern to a female-specific pattern; and (4) *Sxl* downregulates the amount of FNE protein in XX female heads. The rescue of the effect of *Sxl* mutations by a *Sxl* minigene both at the level of *fne-a* and *fne-b* splicing and at the level of FNE protein demonstrates that the effects are specific to *Sxl*. Our data thus show that equal FNE protein abundance in male and females is the surprising outcome of complex regulation at multiple molecular levels.

## Discussion

### Transcript level fluctuations can occur independently of splicing regulation and with no impact on protein levels

Large-scale transcriptomic and proteomic analyses have contributed to the recognition of posttranscriptional regulation in the tuning of protein levels (Vogel and Marcotte 2012). In the case of *fne*, we measured distinctly higher transcript levels in CS males, although FNE protein levels are unchanged between CS males and females (and other examined genotypes aside from the *Sxl^−^* pseudomales). However, in both sexes and in all genotypes (but not in *Sxl^−^* pseudomales), we observed that the relative abundance of mutually exclusive alternative splice forms remains constant, in agreement with independent regulation of splicing and stable transcript levels. The transcript level fluctuations possibly reflect background differences. Thus, to evaluate potential splicing regulation in mutants, we focused on the ratios of alternative isoforms rather than on levels of individual transcripts.

### Identification and regulation of genes expressed differentially in females and males

The *Drosophila* sex determination hierarchy is the classical model of developmentally regulated alternative splicing. To identify genes expressed differentially in males and females, we chose to work with head samples, thereby eliminating large numbers of events restricted to gonadal differentiation. Moreover, the neurons, enriched in heads, are the site of extensive regulation at the level of alternative splicing (Calarco *et al.* 2011; Zheng and Black 2013; Brown *et al.* 2014).

In addition to *dsx* and *fru*, canonical regulators of *Drosophila* sex determination, we identified and further characterized the expression of *fne* and *tango13* as genes expressed in a sex-biased manner. We found that *tango13* sex-specific expression responds to *tra*, *tra2*, and *Sxl* mutations in females as expected if under the control of the canonical sex determination pathway. An intriguing feature is the absence of reciprocity in the regulation of the mutually exclusive *tango13-a* and *tango13*-*b* splice forms, because *tango13-a* levels are reduced in *tra*, *tra2*, and *Sxl* mutants, but *tango13-b* levels are not. This observation suggests that *tra* and *tra2* could possibly have an impact on the levels/stability of the *tango13-a* transcript rather than on the alternative splicing of *tango13* RNA *per se*. The impact of *tra/tra2* alleles on the expression of *tango13-a* is similar to that on the expression of *dsx-F*, consistent with regulation downstream of the sex determination pathway.

### Sex-biased alternative splicing of *fne* and FNE protein levels are both dependent on *Sxl* but independent of *tra/tra2*

In contrast to *fru*, *dsx*, and *tango13*, the expression of *fne* is independent of TRA and TRA2. Crucially, *fne* splicing nevertheless depends on *Sxl* function in female heads: *Sxl^−^* pseudomales switch to a male mode of *fne* alternative splicing, consistent with a role for SXL in promoting, directly or indirectly, the formation of the *fne-b* isoform at the expense of *fne-a* in normal females. Further, although *fne* alternative splicing is male-like in XX *Sxl* pseudomales, FNE protein levels are also upregulated two-fold to three-fold compared to CS males and females. Both male-like splicing and increased FNE protein levels in the pseudomales are reverted by the introduction of a *Sxl^+^* minigene, confirming the specificity of *Sxl* in the control of both the splicing and protein levels. Our data thus show that a *Sxl*-dependent, *tra/tra2*-independent mechanism regulates *fne* expression in females.

### Complex sex-specific *fne* regulation involving multiple molecular levels leads to an equal amount of FNE protein in males and females

CS female and male pools of *fne* RNA yield similar amounts of FNE protein in the two sexes. However, XX *Sxl^M1,fΔ33^* / *Sxl^f7M1^* pseudomales have a male-like pool of *fne* RNA and two-fold to three-fold increased FNE protein levels compared to CS. Because *fne* is an X-linked gene, its expression is presumably influenced by the canonical dosage compensation pathway, which could be responsible for the upregulation of FNE levels in XX *Sxl^−^* pseudomales. However, according to the canonical model, higher *fne* transcript levels would be expected in pseudomales than in males and females, but that is not the case. Additional mechanisms must be at play.

First, increased FNE protein levels in XX *Sxl^−^* pseudomales compared to wild-type males do not result from increased transcript levels. Because males and pseudomales share similar spliced pools of *fne* RNA, their distinct FNE outputs necessarily result from a regulatory mechanism that operates independently of the effects of *Sxl* on alternative splicing. Formally, this mechanism appears to stimulate the translation of *fne* transcripts in XX individuals. Second, increased FNE protein levels, concomitant with changes in alternative splicing but not associated with changes in transcript levels, as in XX *Sxl^−^* pseudomales compared to wild-type females, are consistent with the existence of a *Sxl*-dependent mechanism that downregulates FNE protein levels in XX females. Only the XX-dependent upregulation would persist in *Sxl^−^* pseudomales, hence their increased FNE level. It is conceivable that *fne* regulation by *Sxl* occurs via direct binding of *Sxl* to *fne* transcripts (see File S4). An interesting alternative as a means to regulate its splicing is the possibility that the impact of *Sxl* on *fne* expression occurs indirectly (possibly via an hormonal axis), since the extensive impact of the germline on the expression of somatic genes has been documented (Parisi *et al.* 2010).

### Regulation of *fne* by SXL

*fne* encodes an RNA-binding protein concentrated in the soma of neurons and present throughout development (Samson and Chalvet 2003). It is necessary for the normal development of the mushroom bodies of males and females, and it is involved in the regulation of male courtship (Zanini *et al.* 2012). It is intriguing that the expression of pan-neuronal *fne* is regulated in a sex-biased manner under the control of *Sxl*.

In addition to its role in the development of the germline, *Sxl* is involved in several regulatory pathways in the soma. It responds to a cell autonomous signal (number of X chromosomes) and is crucial both for the sexual development of somatic cells and for dosage compensation in males. SXL, but not TRA or TRA2, is also required independently of the somatic sex determination pathway for the development of a subset of sexually dimorphic neurons, with consequences on female ovulation (Evans and Cline 2013). Additional phenotypes independent of the canonical somatic sex determination pathway but dependent on *Sxl* are the control of the sexually dimorphic body size of flies (Cline and Meyer 1996) and the sex-specific bristle number on the A5 sternite (Penn and Schedl 2007). The latter occurs through general downregulation of the *Notch* pathway by SXL in multiple tissues (Penn and Schedl 2007). Thus, the *Sxl*-regulated expression of *fne* fits within the context of *Sxl* acting in parallel with the canonical *Sxl-tra/tra2* cascade, constituting an example of its impact on tissues that do not show obvious sexual dimorphism.

### What impact for the *Sxl*-dependent regulation of *fne*?

*fne* is a member of a fairly new multigene family restricted to dipterans (Samson 2008). The birth of this family predates the role of SXL in sex determination, which is restricted to the drosophilids (Meise *et al.* 1998). Based on our RNA-Seq data and the Flybase models (St Pierre *et al.* 2014), sex-specific alternative splicing has not been reported for either of the other two paralogues in this family, *elav* (*embryonic lethal abnormal visual system*, X linked) or *rbp9* (*RNA binding protein 9*, second chromosome). *elav* is the result of a retrotransposition and is likely to have acquired new *cis*-regulatory elements in the process (Samson 2008). It autoregulates via a posttranscriptional mechanism involving its 3′ UTR (Samson 1998). It is unclear whether the *Sxl*-dependent regulation of *fne* is an ancestral property that has been lost for *rbp9* or was recently acquired. Nevertheless, sex-specific alternative splicing provides *fne* with the ability to be differentially regulated in females, which may have an important impact on sex-specific nervous system function or development, for which there are numerous instances of a role for *Sxl*. Within the context of the canonical sex determination pathway, *Sxl* regulates the expression of *fru* and *dsx*, two transcription factors crucial for behavior and nervous system function. SXL also controls, via an independent pathway, specific aspects of female behavior (Evans and Cline 2013). Still outside of the context of the canonical sex determination pathway, *Sxl* regulates the neurogenic locus *Notch* (Penn and Schedl 2007). Further, in *Drosophila virilis*, SXL protein accumulates in the male developing nervous system, consistent with a role there (Bopp *et al.* 1996). Thus, the control exerted by *Sxl* on pan-neuronal *fne* outside of the context of the canonical sex determination pathway may be part of the heritage of an SXL ancestral function more focused on the nervous system than on sexual differentiation.

## 

## Supplementary Material

Supporting Information
