# Supplementary material for: Sxl-Dependent, tra/tra2-Independent Alternative Splicing of the Drosophila melanogaster X-Linked Gene found in neurons
Source: G3 (Bethesda). 2015 Oct 26;5(12):2865–74. doi: 10.1534/g3.115.023721 (PMC4683657; doi:10.1534/g3.115.023721)
Supplement: Supporting Information [file supp_5_12_2865__index.html]

Sxl-Dependent, tra/tra2-Independent Alternative Splicing of the Drosophila melanogaster X-Linked Gene found in neurons — Supporting Information 

# *Sxl*-Dependent, *tra/tra2*-Independent Alternative Splicing of the *Drosophila melanogaster* X-Linked Gene *found in neurons*

## Supporting Information for Sun *et al.*, 2015

**Files in this Data Supplement:**

- Figure S1 - Graphic representation of sex-biased alternative splicing from the RNA-Seq analysis. RPKM for each alignment track are shown as bar graphs (female: red; male: blue). Arcs denote splice junctions, their thickness corresponds to the number of reads spanning the junctions. The introns and exons are compressed with different ratios (15:2, as default). Gene annotation tracks are shown at the bottom, and arrows in introns represent the direction of transcription. For key splice events that have been also evaluated by qPCR, relevant exons and arcs (splice junctions) are in red (female-enriched junction) or blue (male-enriched junction). The significance of each splicing event (females vs males) was tested by using high quality reads identified by Spanki. Significance levels are marked "\*", "\*\*", and "\*\*\*" to respectively indicate q<0.05, q<0.01, q<0.001. (.pdf, 2.71 MB)
- File S1 - Complete Spanki analysis of the RNA-Seq data. (.xlsx, 3.4 MB)
- File S2 - Statistical analysis details of qPCR results. (.xls, 119 KB)
- File S3 - Statistical analysis details of the enrichments. (.xls, 459 KB)
- File S4 - Supplemental discussion. (.docx, 128 KB)
- Table S1 - Spanki analysis of the RNA-Seq data and qPCR validation. (.xlsx, 57 KB)
- Table S2 - Primer list with their properties. (.xlsx, 12 KB)
