# Supplementary figures and images for: Sxl-Dependent, tra/tra2-Independent Alternative Splicing of the Drosophila melanogaster X-Linked Gene found in neurons
Source: G3 (Bethesda). 2015 Oct 26;5(12):2865–74. doi: 10.1534/g3.115.023721 (PMC4683657; doi:10.1534/g3.115.023721)

\* dsx

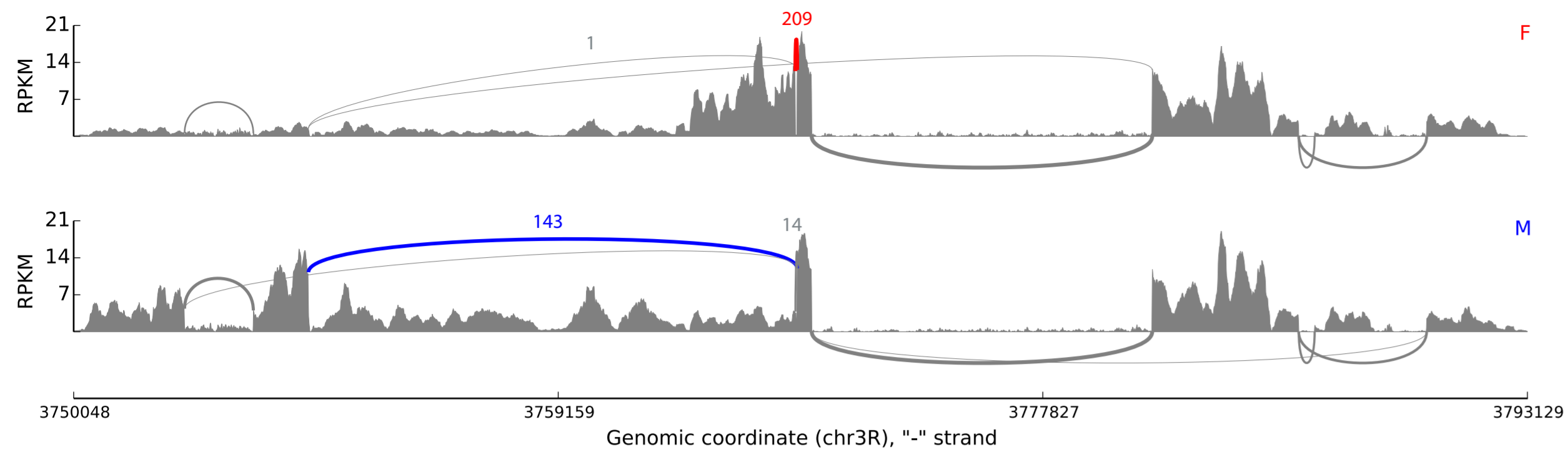

\* fru

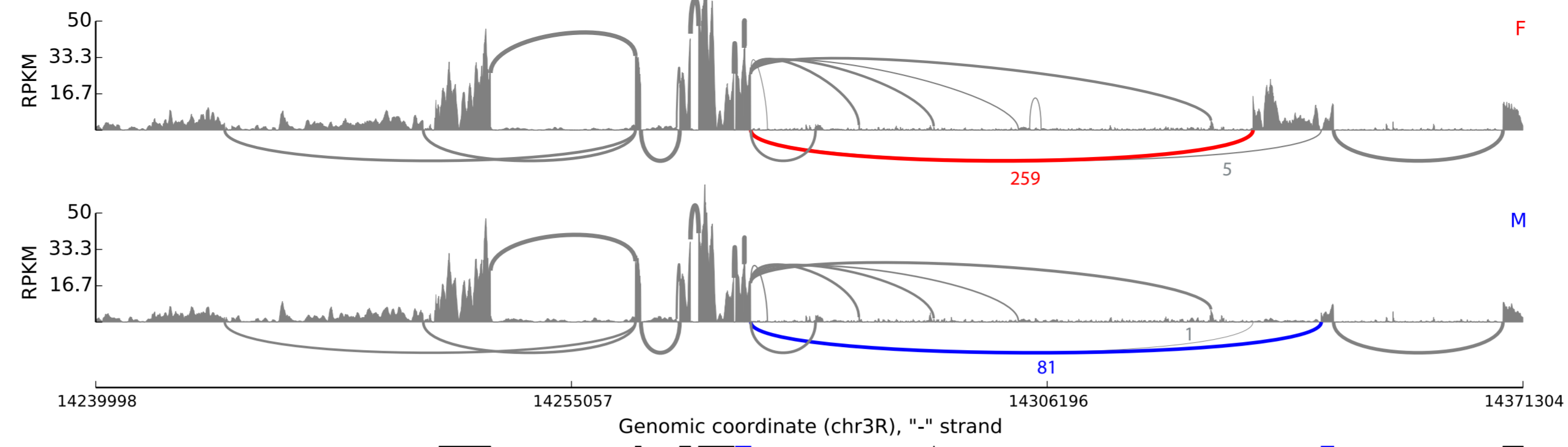

\* Sxl

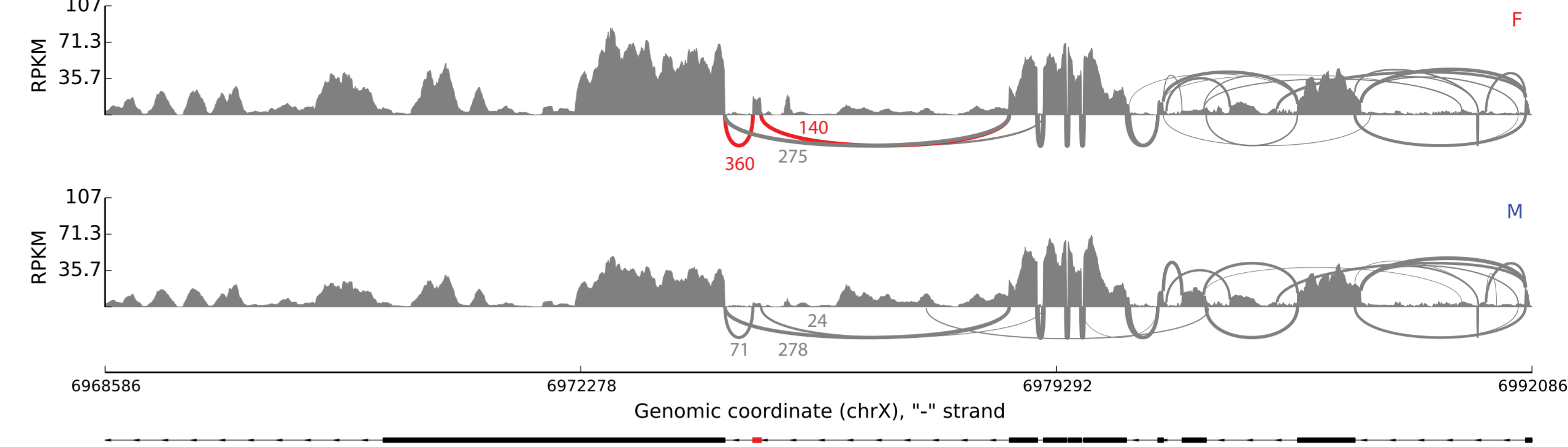

tango13

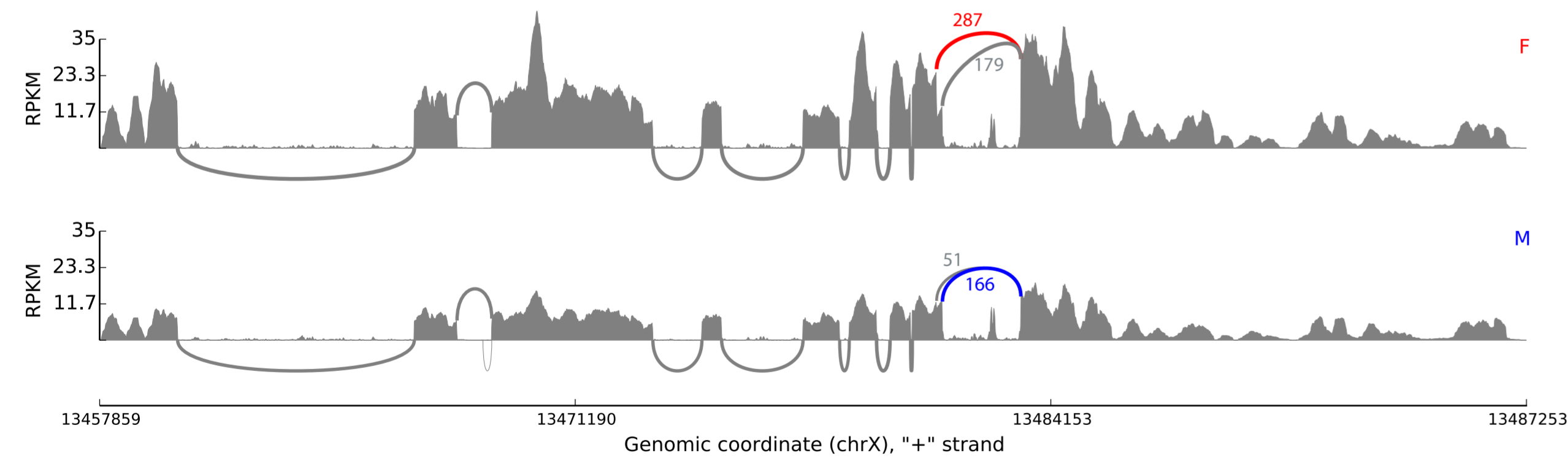

regucalcin

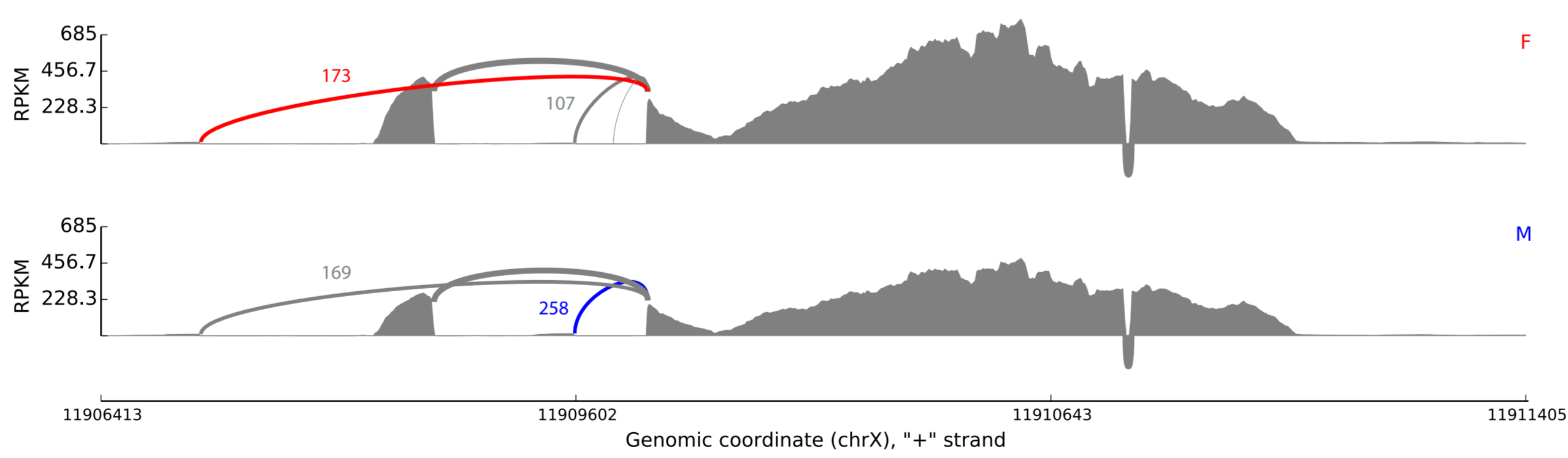

\* fbp

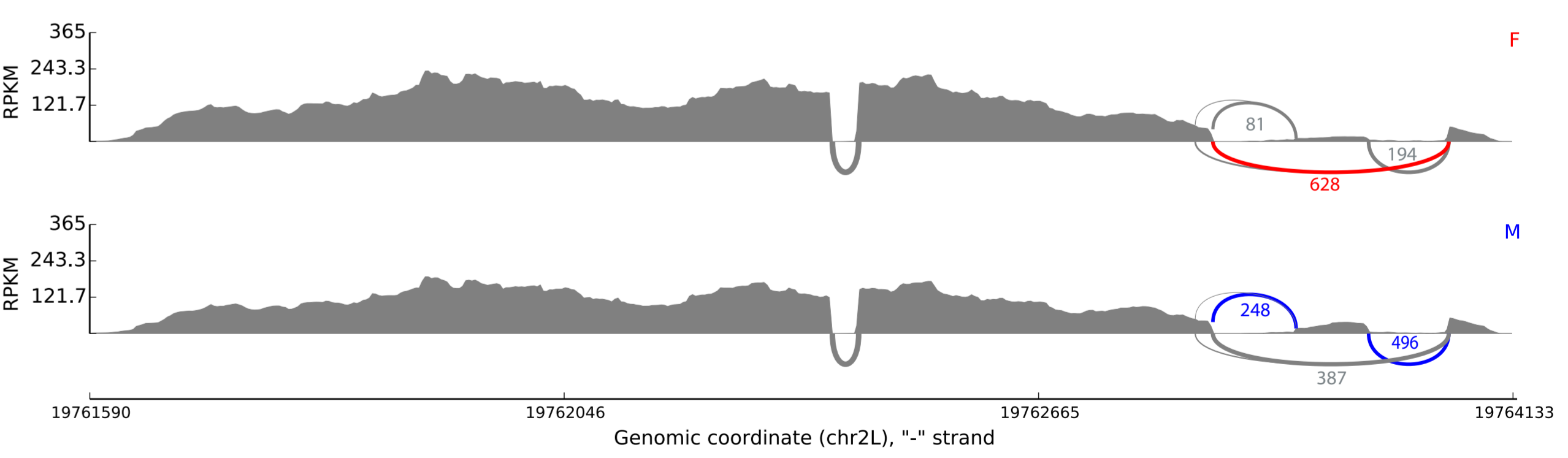

Supplement: Supporting Information [file supp_g3.115.023721_Figure_S1.pdf]
