# Supplementary material for: Sxl-Dependent, tra/tra2-Independent Alternative Splicing of the Drosophila melanogaster X-Linked Gene found in neurons
Source: G3 (Bethesda). 2015 Oct 26;5(12):2865–74. doi: 10.1534/g3.115.023721 (PMC4683657; doi:10.1534/g3.115.023721)
Supplement: Supporting Information [file supp_g3.115.023721_File_S4.docx]

**File S4 Supplemental discussion**

**Is SXL directly binding *fne* transcripts?**

SXL's impact on *tra* and *msl-2* splicing in the canonical somatic sex-determination pathway has been extensively investigated. *Sxl* also acts in the cytoplasm to inhibit the translation of *msl2* transcripts in females, encoding a key regulator of dosage compensation in males. Two other genes also have their regulation dependent upon direct binding of SXL to their transcripts: first, *nanos*, necessary for the renewal of adult female germline stem cells, is down regulated by *Sxl* in daughter cells committed to differentiation. SXL directly binds *nanos* transcripts. In the absence of SXL, Nanos protein continues to be produced in the germ cells (Chau et al. 2009). Second, SXL negatively regulates *Notch* protein levels in many female tissues, including the ovaries, wings and sternites. This regulation does not depend upon *tra* and *tra2*. Crosslinking of SXL and *Notch* RNA performed in ovarian extracts demonstrated direct interaction (Penn and Schedl 2007).

We do not exclude direct binding of SXL to *fne* pre-mRNA. Experimental and consensus SXL binding sites are U-rich sequences (Sosnowski et al. 1989; Horabin and Schedl 1993b; Horabin and Schedl 1993a; Valcárcel et al. 1993; Bashaw and Baker 1997; Kelley et al. 1997; Gawande et al. 2006; Li et al. 2009; Ray et al. 2013). Predicting SXL targets and the regulatory impact of SXL binding is difficult because U-rich sequences are common in untranslated regions, and that SXL protein has multiple documented functions, at the levels of splicing, translational repression, or 3' UTR formation, involving distinct mechanisms both in the cytoplasm and the nucleus. In the case of *fne*, two mechanistically distinct presumptive scenarios could explain our data. The sequence motif UUUUUUAUCUCUUUUU lies 17 nucleotides downstream of the 5’ splice site generating *fne-a* transcripts and 159 nt upstream of the 5’ splice site generating *fne-b* transcripts (Figure 1). This sequence is thus retained in mature *fne-b* transcripts, but not in those of *fne-a*. Binding of SXL to this site in the primary transcript could conceptually mask the nearby *fne-a* 5’ splice site and cause preferential use of the downstream *fne-b* 5’ splice site in females, but not in males and *Sxl* XX pseudo-males. Alternatively, an intronic UUUUUUUU sequence is positioned 403 nt downstream of the *fne-a* 5’ splice site and only 204 nt downstream of the *fne-b* 5’ splice site (Figure 1). If SXL can bind this site, it could possibly recruit factors to the *fne-b* 5’ splice site, but not to the more distant *fne-a* site.

**A possible mechanism for Sxl-dependent down regulation of fne in females**

In females, where SXL-regulated splicing of *fne* occurs, the *fne-b* transcript is enriched, relative to its levels in males. *fne-b* transcripts include 199 nucleotides that are spliced out from *fne-a* transcripts. Structural differences in the 5' UTR can modulate translation efficiency (Araujo et al. 2012). Although we do not known whether this applies to *fne-a* and *fne-b*, it suggests a possible mechanism for *Sxl*-dependent down regulation of *fne* in females. It is conceivable that there is reduction (or blocking) of the efficiency of translation of female-enriched *fne-b* transcripts, perhaps through binding of SXL itself since the UUUUUUAUCUCUUUUU potential SXL binding site is present specifically in *fne-b* but not *fne-a*. This would be reminiscent of the *Sxl*-dependent regulation of *msl-2* where intronic SXL binding blocks *msl2* splicing, while 3’ UTR binding blocks translation (Bashaw and Baker 1997; Kelley et al. 1997).

Araujo, P. R., K. Yoon, D. Ko, A. D. Smith, M. Qiao et al., 2012 Before It Gets Started: Regulating Translation at the 5' UTR. Comp Funct Genomics 2012: 475731.

Bashaw, G. J., and B. S. Baker, 1997 The regulation of the Drosophila *msl-2* gene reveals a function for *Sex-lethal* in translational control. . Cell 89: 789-798.

Chau, J., L. S. Kulnane and H. K. Salz, 2009 *Sex-lethal* facilitates the transition from germline stem cell to committed daughter cell in the Drosophila ovary. Genetics 182: 121-132.

Gawande, B., M. D. Robida, A. Rahn and R. Singh, 2006 (2006) Drosophila *Sex-lethal* protein mediates polyadenylation switching in the female germline. 25:1263-72. EMBO J. 25: 1263-1272.

Horabin, J. I., and P. Schedl, 1993a Regulated splicing of the Drosophila *Sex-lethal* male exon involves a blockage mechanism. Mol. Cell Biol. 13.

Horabin, J. I., and P. Schedl, 1993b *Sex-lethal* autoregulation requires multiple cis-acting elements upstream and downstream of the male exon and appears to pivot primarily on the male exon 5' splice site. Mol. Cell Biol. 13: 7734-7746.

Kelley, R., J. Wang, L. Bell and M. Kuroda, 1997 *Sex lethal* controls dosage compensation in Drosophila by a non-splicing mechanism. Nature 387: 195-199.

Li, H., H. B., W. A., F. T., Ruan J et al., 2009 The Sequence Alignment/Map format and SAMtools. Bioinformatics 25: 2078-2079.

Penn, J. K. M., and P. Schedl, 2007 The master switch gene *Sex-lethal* promotes female development by negatively regulating the N signaling pathway. Dev Cell 12: 275-286.

Ray, D., H. Kazan, K. B. Cook, M. T. Weirauch, H. S. Najafabadi et al., 2013 A compendium of RNA-binding motifs for decoding gene regulation. Nature 499: 172-177.

Sosnowski, B. A., J. M. Belote and M. McKeown, 1989 Sex-specific alternative splicing of RNA from the *transformer* gene results from sequence-dependent splice site blockage. Cell 58: 449-459.

Valcárcel, J., R. Singh, P. Zamore and G. MR, 1993 The protein Sex-lethal antagonizes the splicing factor U2AF to regulate alternative splicing of transformer pre-mRNA. Nature 362: 171-175.
